# Metabolic symbiosis and competition: the dual nature of TAM-tumor cell cross-talk in tumor progression

**DOI:** 10.3389/fonc.2026.1821192

**Published:** 2026-04-22

**Authors:** Chaokai Ba, Shizheng Tong, Zefan Wang, Huiting Zhu, Shenxian Qian

**Affiliations:** 1 The Fourth School of Clinical Medicine, Zhejiang Chinese Medical University, Hangzhou, China; 2 Hangzhou First People’s Hospital, Westlake University, Hangzhou, China

**Keywords:** cancer, hypoxia, metabolic reprogramming, tumor associated macrophages, tumor microenvironment

## Abstract

Cancer cells and tumor associated macrophages (TAMs) engage in a sophisticated metabolic symbiosis within the tumor microenvironment (TME), where reciprocal metabolite exchange drives immune evasion and malignant progression. This review posits that TAMs functional plasticity is not merely a consequence but a driver of tumor fitness, governed by extensive metabolic rewiring. We dissect the mechanistic underpinnings of this “metabolic dialogue,” focusing on the convergence of glycolytic flux, the lactate shuttle, amino acid catabolism, lipid reprogramming, hypoxia-induced adaptations, and TCA cycle anaplerosis. Beyond delineating these pathways, we critically evaluate emerging therapeutic paradigms that target these metabolic nodes, advocating for precision interventions capable of disrupting this pro-tumorigenic alliance while restoring immune surveillance.

## 1 Introduction

The increasing aging of the global population, rapid socio-economic development, and worsening environmental pollution have led to a growing burden of cancer, making it the leading cause of death worldwide. Although conventional treatment methods such as surgery, chemotherapy, and radiotherapy have made significant progress in the diagnosis and treatment of early cancer, the survival rate of patients with advanced or metastatic malignant tumors is still unsatisfactory. This is mainly due to the fact that these therapies cannot specifically target the tumor microenvironment (TME) (1, 2).

Tumor associated macrophages (TAMs) are the dominant group of tumor-promoting immune cells in TME, accounting for 30% -50% of the total number of tumor infiltrating immune cells (3). Functionally, TAMs mediate immune suppression through signaling pathways such as transforming growth factor - β (TGF - β) and interleukin-10 (IL-10), thereby promoting immune escape. In addition, the vascular endothelial growth factor (VEGF) secreted by TAMs not only promotes tumor angiogenesis, but also protects tumor cells from oxidative stress caused by chemotherapy, endowing them with chemotherapy resistance, ultimately leading to the formation of chemotherapy resistance (4–6).

The function of TAMs exhibits stage specific and tissue-specific characteristics, regulated by their origin, microenvironmental signals, and intracellular pathways (7). Macrophages can be roughly divided into two polarization states based on surface marker expression, cytokine profile, and metabolic characteristics: pro-inflammatory (M1-like) phenotype, which is the classical activation phenotype; Anti-inflammatory (M2-like) phenotype, i.e. alternative activation phenotype (8). It is worth noting that macrophages exhibit significant phenotypic plasticity, and their metabolic reprogramming is closely related to functional differentiation (9).

Metabolic reprogramming is a hallmark of cancer that extends to the TME, critically shaping immune cell function. Emerging evidence indicates that the metabolic plasticity of TAMs is intrinsically linked to their phenotypic polarization, where distinct metabolic signatures drive either pro-inflammatory (M1-like) or immunosuppressive (M2-like) states (10–13). Specifically, the TME forces—such as hypoxia and nutrient scarcity—rewire TAMs metabolism, fostering an immunosuppressive landscape that supports tumor progression (14, 15). While the general principles of this metabolic coupling are becoming clear, the specific molecular mechanisms governing glucose, lipid, and amino acid fluxes between tumor cells and TAMs remain complex and context-dependent.

This article explores the dual role of TAMs metabolic reprogramming in tumor progression, with a focus on pathways of glucose, lipid, and amino acid metabolism, as well as hypoxia and lactate signaling. Meanwhile, this article also evaluates the potential and challenges of tumor treatment strategies.

## 2 Characteristics of TAMs

### 2.1 Origin of TAMs

Macrophages are larger phagocytic cells that exhibit specific differences in tissue structure and function. Typical examples include Kupffer cells in the liver, alveolar macrophages in the lung system, microglia in the central nervous system, and osteoclasts in bone tissue (16, 17). In the field of cancer, TAMs mainly come from circulating monocytes (Ly6C+) from the bone marrow, which are recruited to the tumor site and differentiate there (18). Recent studies employing single-cell transcriptomics and epigenetic profiling have established that tissue-resident macrophages (TRMs) constitute a significant source of TAMs. During tumorigenesis, TRMs originating from normal tissues undergo phenotypic conversion into TAMs while retaining partial transcriptional signatures characteristic of their tissue-of-origin.

### 2.2 TAMs polarization and plasticity

The current understanding of macrophage polarization stems from the groundbreaking M1/M2 classification model proposed by Mills et al. in 2000 (19).This dichotomy reflects the dual role played by macrophages in tumor progression, exhibiting both anti-tumor (M1-like) activity and pro-tumor (M2-like) activity. TAMs have significant plasticity and can switch between M1-like and M2-like phenotypes in the TME. Numerous studies have confirmed that TAMs are regulatory factors in tumor metastasis, affecting almost every step of the metastasis cascade reaction (20). Their functional diversity in the TME makes them regulatory factors for tumor inhibition and promotion.

M1-like TAMs exert strong anti-tumor effects through cytotoxic effects mediated by reactive oxygen species (ROS) and nitric oxide (NO), secretion of pro-inflammatory cytokines (such as IL-1 β, IL-6, IL-12, IL-23, TNF-α), and recruitment of immune effector cells, thereby enhancing their overall pro-inflammatory and anti-tumor characteristics (16, 21).

**Figure 1:**
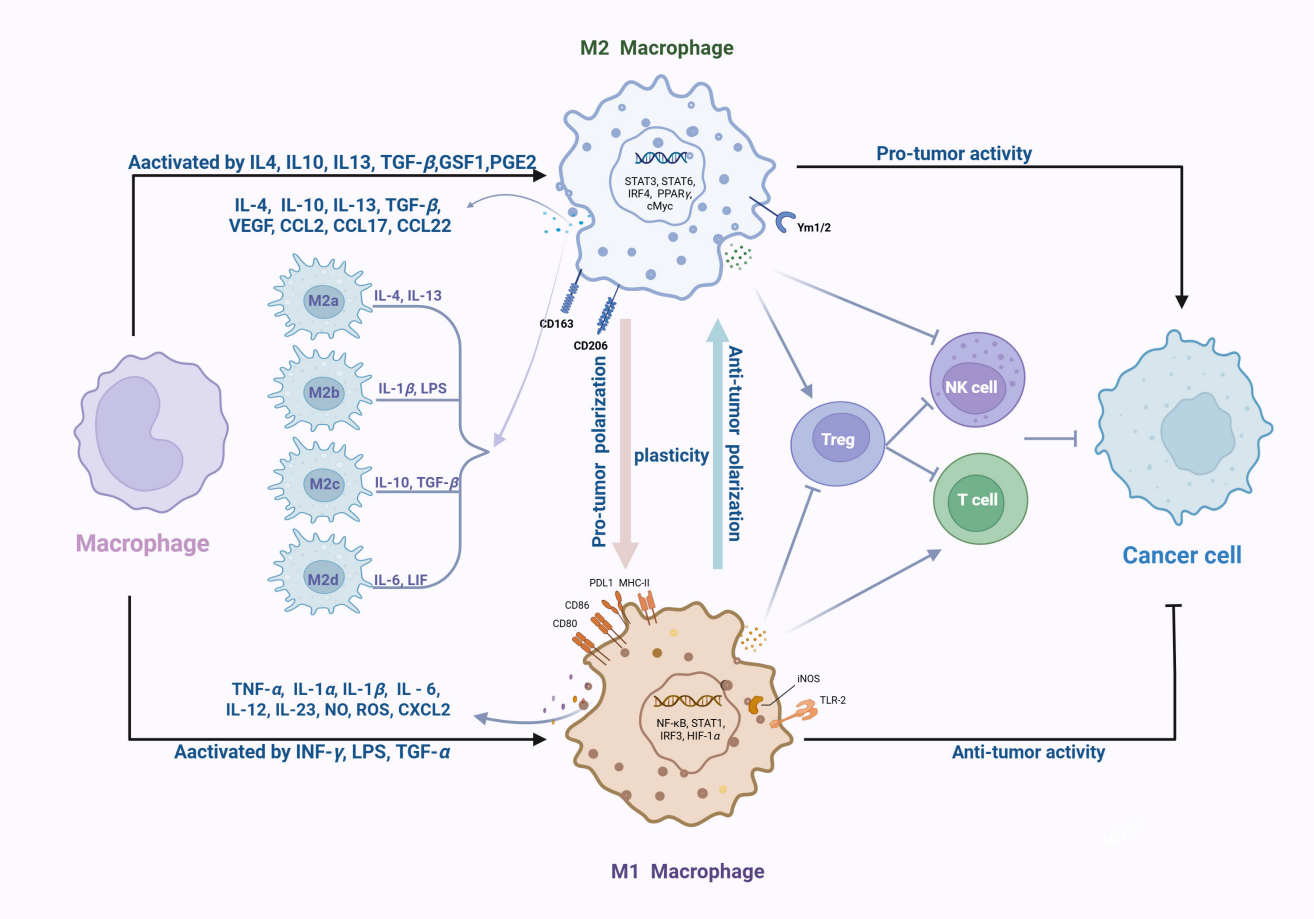
Polarization and functional characteristics of TAMs. The diagram illustrates the extracellular stimuli and transcription factors driving macrophage polarization into M1-like or M2-like (M2a-d) phenotypes. It outlines their respective secretory profiles: pro-inflammatory mediators from M1-like TAMs, and anti-inflammatory/angiogenic factors from M2-like TAMs. Additionally, the network depicts M2-like macrophages inhibiting effector T cells and NK cells while promoting Tregs, whereas M1-like macrophages target cancer cells.

In contrast, M2-like TAMs promote tumor development through different pathways: (1) secreting anti-inflammatory cytokines (IL-4, IL-10, IL-13) after being stimulated by Th2 signaling; (2) Expressing unique surface markers (CD163, CD206, CD209) and chemokines (CCL2); (3) Inducing immune tolerance through type 2 reactions. These mechanisms collectively form an immunosuppressive TME that is conducive to angiogenesis, tumor growth, and metastasis and diffusion (22, 23).The M2-like phenotype can be further divided into four different subtypes based on activation stimuli: M2a (activated by IL-4/IL-13), M2b (activated by immune complex binding Toll like receptor ligands and IL-1 β), M2c (activated by glucocorticoids/IL-10/TGF-β), and M2d (activated by IL-6/adenosine), each subtype having a unique cytokine/chemokine expression profile (24–26). Although M2a and M2b macrophages primarily mediate Th2 type immune responses, M2c and M2d subgroups play a critical role in immune suppression and tissue remodeling (27–29) (**Figure 1**).

TAMs mainly exhibit a polarization phenotype similar to the M2-like type, which contributes to the development of tumors in the TME, with most exhibiting pro-tumor properties (18, 30). Compared with M1-like TAMs, M2-like TAMs express anti-inflammatory cytokines, phagocytic receptors, angiogenic factors, and proteolytic enzymes (31).

Therefore, the presence of M1-like TAMs is positively correlated with good clinical prognosis in solid tumors. On the contrary, the increased infiltration of M2-like TAMs is strongly positively correlated with enhanced angiogenesis, metastatic progression, and poorer clinical outcomes in various types of cancer (32–36).

### 2.3 Functional roles of TAMs in the TME

TAMs are pivotal orchestrators throughout the tumor lifecycle, driving processes from initiation to therapy resistance. They foster immune evasion by suppressing T-cell activity, stimulate angiogenesis to fuel tumor growth, and remodel the extracellular matrix to enable invasion (37).

Their functional dominance stems largely from a prevalent M2-like polarization, characterized by high expression of anti-inflammatory cytokines, phagocytic receptors, and pro-angiogenic and proteolytic factors (37). This phenotype supports tumor progression and metastasis (38).

Despite this bias, TAMs exhibit significant plasticity; their state can shift dynamically in response to microenvironmental cues (38, 39). They actively contribute to metastatic spread by preparing distant sites—via ECM degradation, induction of EMT, and facilitating tumor cell entry into circulation (40).

Critically, their pro-tumor functions are sustained by metabolic reprogramming: under hypoxia and nutrient stress, TAMs rewire glucose, lipid, and amino acid metabolism to maintain energy homeostasis and reinforce their M2-like, tumor-promoting identity (41).

## 3 Metabolic coupling between tumor cells and TAMs

To maintain rapid growth and viability within the harsh TME—characterized by hypoxia, nutrient scarcity, and hostile conditions—malignant cells develop sophisticated metabolic adaptations. Such metabolic rewiring not only interferes with intracellular signaling cascades but also substantially reshapes the TME via abnormal metabolite buildup (42).

During the 1920s, Otto Warburg made the pioneering observation that tumor tissues display markedly elevated glucose consumption and lactate release, even in the presence of adequate oxygen, resulting in extracellular acidification—now termed the Warburg effect. Whereas normal cells primarily generate adenosine triphosphate (ATP) via mitochondrial OXPHOS, cancer cells preferentially rely on aerobic glycolysis for energy production, regardless of oxygen availability (43, 44). Such metabolic rewiring is characterized by three hallmarks: enhanced glycolytic flux, suppressed mitochondrial OXPHOS function, and heightened lactate output (45, 46). Notably, this altered metabolic landscape critically influences the phenotypic and functional properties of TAMs.

Comparable to tumor cells, diverse TAMs subpopulations display marked dependence on aerobic glycolysis, despite divergent regulatory circuits and functional outputs. In the TME, glucose deprivation and lactate enrichment have evolved beyond simple metabolic barriers to become essential directional signals governing TAMs polarization toward an M2-like immunoregulatory phenotype. For example, lactate helps stabilize HIF-1α, thereby upregulating the expression of pro-tumor mediators such as VEGF and arginase-1 (Arg-1) in TAMs (47, 48). At the same time, in order to save glucose for cancer cells to utilize, TAMs adapts to metabolism by enhancing the acquisition and breakdown metabolism of alternative substrates such as fatty acids and glutamine. This reprogramming towards oxidative metabolic pathways further consolidates its immunosuppressive ability in TME.

**Figure 2:**
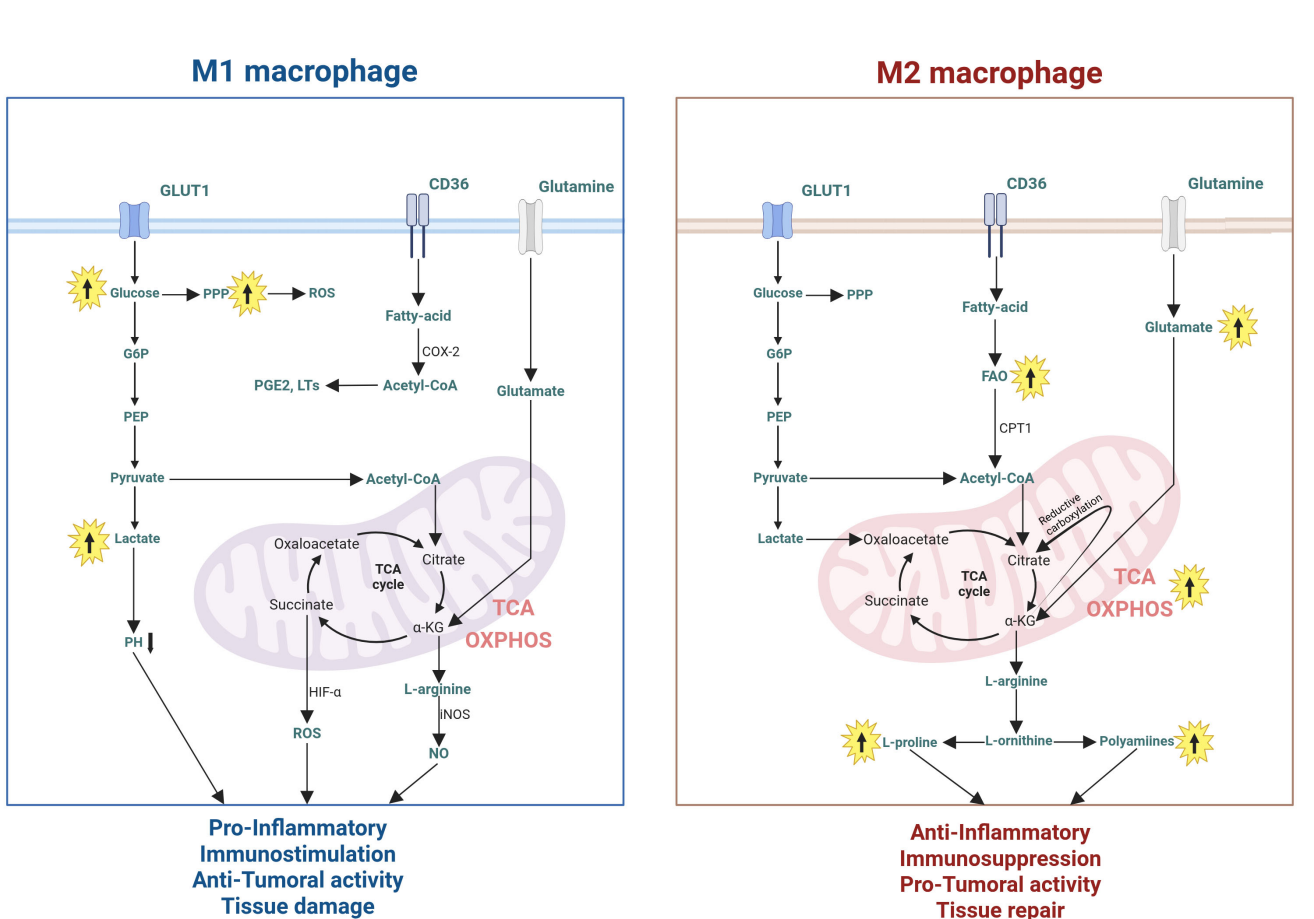
Metabolic phenotypes are context-dependent and may overlap in the TME. M1-like and M2-like macrophage exhibit distinct metabolic profiles. M1-like macrophage primarily rely on glycolysis for energy production, whereas M2-like macrophage predominantly utilize fatty acid oxidation and oxidative phosphorylation.

Comprehensive multi omics analysis has confirmed that TAMs consistently upregulate metabolic pathways, including glycolysis, fatty acid synthesis (FAS), and FAO, and undergo significant glutamine metabolic reprogramming. The mouse model revealed significant metabolic differences between polarized macrophage subtypes: M1-like macrophages primarily utilize glycolysis to generate energy and exhibit enhanced reactive ROS generation ability, while M2-like macrophages preferentially utilize glutamine breakdown and FAO to meet their bioenergy needs (49) (**Figure 2**).

## 4 Metabolic changes of TAMs

### 4.1 Glucose metabolism

As mentioned earlier, TAMs are the most common immune infiltrating cells in the TME and exhibit significant metabolic dependence on aerobic glycolysis. However, it must be emphasized that the driving factors and functional consequences behind this metabolic preference are completely different from those in malignant cells. In the nutrient deficient TME, limited glucose supply and elevated lactate concentration have surpassed their traditional role as metabolic stressors and become powerful directive signals, actively regulating the polarization and functional programming of TAMs. A large amount of evidence shows that TAMs can not only increase the glycolysis flux of tumors, but also promote malignant progression in a variety of cancers, including breast cancer, bladder cancer, non-small cell lung cancer and hepatocellular carcinoma (50, 51).

M1-like TAMs exhibit enhanced aerobic glycolysis and pentose phosphate pathway (PPP) activity, enabling effective activation and cytokine secretion. However, long-term glucose exposure can impair the phagocytic function of M1-like TAMs (52, 53).

M2-like TAMs mainly relies on mitochondrial metabolism through the TCA cycle, upregulates OXPHOS and adipose tissue FAO, thereby promoting immunosuppressive function, inhibiting anti-tumor immunity, and promoting tumor metastasis (54). Glucose, as a metabolic substrate, promotes FAS, thereby supporting the enhancement of FAO in M2-like TAMs. This process connects glycolysis, FAO, and adipogenesis to form a comprehensive metabolic network (55).

The uptake of glucose also promotes various post-translational modifications. O-GlcNAcylation is an important glycosylation modification that serves as a vital nutrient sensor and regulatory switch within cells, dynamically controlling protein function in response to metabolic cues (56). An increase in glucose uptake enhances the activity of the Hexosamine Biosynthesis Pathway (HBP), thereby promoting O-GlcNAcylation of Cathepsin B(CTSB) in TAMs. This modification helps CTSB to be secreted from the TME, ultimately promoting tumor metastasis and the formation of chemotherapy resistance (54). Specifically, TAMs stimulate O-GlcNAcylation of lysosomal protease B, which subsequently promotes cancer metastasis and chemotherapy resistance (54). It is worth noting that several Inhibitors of O-GlcNAcylation have been shown to inhibit cancer cell growth and increased sensitivity to chemotherapy drugs (57–59).

Glucose metabolism reprogramming can dynamically regulate immune function, providing a theoretical basis for metabolic immune targeted therapy. Specifically, the N6-methyladenosine (m6A)-modified circular RNA QSOX1 (circQSOX1, derived from the quiescin sulfhydryl oxidase 1 gene) interacts with and regulates phosphoglycerate mutase 1 (PGAM1), a glycolytic enzyme, thereby enhancing glycolysis in colorectal cancer cells and promoting immune escape (60). The anti-tumor effect mediated by HKB99 (a PGAM1 inhibitor) requires a simultaneous reduction in the infiltration of TAMs and an increase in the recruitment of CD8+T cells (61).

Extracellular vesicles from TAMs drive the reprogramming of glycolysis in tumor cells. Specifically, extracellular vesicles secreted by M2-like TAMs activate the β - catenin and HIF-1α signaling pathways, thereby enhancing aerobic glycolysis in gastric cancer cells and promoting tumor progression (62). In hepatocellular carcinoma, TAM-derived exosomes promote the transfer of M2-like macrophage polarization associated long non-coding RNA (lncMMPA) to tumor cells, thereby promoting aerobic glycolysis and enhancing the proliferation ability of tumor cells (63).

In addition, TAMs can enhance OXPHOS activity, promote ATP production, and maintain the tricarboxylic acid (TCA) cycle function (64). Specifically, IL-4-induced M2-like TAMs upregulates OXPHOS in the TME. This metabolic reprogramming plays a role in promoting M2-like polarization and mediating its immunosuppressive function (65, 66).

In summary, these metabolic shifts make glycolysis a promising therapeutic target, inhibitors such as 2-DG and AZD3965 are being evaluated in clinical trials to reprogram TAMs toward an anti-tumor phenotype. By inhibiting glycolytic activity, TAMs may transform into a tumor suppressive phenotype, providing dual benefits: reducing immunosuppressive TME factors while enhancing cytotoxic immune responses. A new metabolic immune regulation strategy targeting macrophage glucose metabolism has been established.

### 4.2 Lactate and acidification

The Warburg effect produces a large amount of pyruvate, which is converted into lactate by enzymes and released into the extracellular space, ultimately forming an acidic microenvironment (low pH). This acidosis promotes angiogenesis and induces chemotherapy resistance, while impairing anti-tumor immunity and driving tumor progression (67–71).

The lactate produced by tumors acts on TAMs through the upregulation of VEGF and ARG1 mediated by HIF-1α, thereby inhibiting immune responses and promoting their polarization towards M2-like type (72–75). The uptake of lactate by MCTs can enhance this process, while extracellular vesicles secreted by TAMs containing HIF-1α protective long non coding RNA promote glycolysis in cancer cells (14, 76). In KRAS mutant colorectal cancer, the stabilization of HIF-1α induces the production of colony-stimulating factor-2 (CSF2), promoting TAMs polarization and cetuximab resistance (77). Lactic acid stabilizes HIF-2α by inhibiting v-ATPase, further enhancing the level of M2-like TAMs with low MHC-II expression (78). In addition, lactate inhibits T cell function through ARG1-mediated arginine depletion (79) and induces histone lactylation to promote immune escape (78), thus becoming the main regulatory factor for tumor immune suppression.

The accumulation of lactate in the TME plays a crucial role in shaping the pro-tumor function of M2-like TAMs. Mechanistically, lactate signaling activates a proton sensitive G protein coupled receptor GPR65 on TAMs, triggering a cascade reaction of cAMP/PKA/CREB signaling and subsequently releasing high mobility group protein 1 (HMGB1), thereby enhancing M2-like polarization (80). In addition, elevated lactate levels activate the ERK-STAT3 signaling axis to drive transcriptional reprogramming of TAMs, promoting the formation of an immunosuppressive microenvironment and facilitating tumor immune escape (81). In lung cancer, lactate induces the transfer of nucleolus and spindle associated protein 1 (NUSAP1) to the nucleus, thereby recruiting JUNB-FRA1-FRA2 transcription complex to the DESMIN promoter and enhancing its transcriptional activity. This lactate NUSAP1-DESMIN axis promotes the activation of cancer associated fibroblasts (CAFs) and the secretion of IL-8, forming a chemotactic gradient that can attract TAMs and drive their M2-like polarization (82).

Overall, these research findings highlight the crucial role of lactate in regulating a self-reinforcing cycle that integrates tumor metabolic dysregulation with immunosuppressive mechanisms. This series of processes locates the lactate driven signaling pathway in TAMs, making it a promising therapeutic target for novel cancer immunotherapy. By regulating the HIF-1α/STAT3 signaling pathway and restoring arginine metabolism by inhibiting ARG1, targeting lactate metabolism can transform TAMs from a pro-tumor phenotype to an anti-tumor phenotype. Inhibition of lactate transporter protein (MCT1/4) or lactate dehydrogenase (LDH) can overcome immune suppression caused by TAMs and work synergistically with immune checkpoint inhibitors.

### 4.3 Lipid metabolism

Tumor cells activate lipid metabolism to meet their bioenergy needs beyond glycolysis (83). In response, TAMs enhances lipid metabolism reprogramming by enhancing fatty acid uptake (CD36/FABP4-mediated) and cholesterol efflux (ABC transporter-dependent), driving immunosuppressive polarization (84, 85). This rewiring links lipid metabolism with immune suppression (via PPAR γ) and chemotherapy resistance (via drug efflux pumps), establishing the lipid pathway as a regulator of TAMs function in cancer.

Compared with normal tissue cells, cancer cells exhibit significant disturbances in lipid metabolism, manifested by enhanced synthesis of new fatty acids and activation of lipid efflux mechanisms. These metabolic changes are particularly critical in TAMs, where lipid accumulation and FAO not only drive their differentiation but also promote pro-tumor function. The receptor CD36 is a regulatory factor for lipid uptake, which is upregulated in TAMs and contributes to immune recognition, inflammatory response, cell adhesion, and apoptosis processes (86). Through the CD36 dependent mechanism, TAMs exhibit enhanced lipid internalization ability, leading to intracellular lipid droplet accumulation and functional polarization (87–89). Mechanistically, CD36 regulates sphingolipid metabolism based on tumor-derived chemokines, promoting monocyte recruitment and subsequent accumulation of TAMs, thereby jointly promoting metastatic progression (90).

In addition, TAMs mainly express fatty acid binding proteins (FABPs) to promote the transport and metabolism of fatty acids. Specifically, FABP4 in TAMs maintains the oncogenic IL-6/STAT3 signaling pathway and enhances its immunosuppressive phenotype (91). In breast cancer, lipid rich TAMs accelerate tumor progression through FABP4-mediated lipolysis and lipid utilization (92). On the contrary, in hepatocellular carcinoma, drug inhibition of FABP5 enhances the efficacy of immunotherapy, highlighting the therapeutic potential of targeting the TAMs lipid metabolism pathway (93).

TAMs accumulate cholesterol due to obstacles in phagocytic clearance or dysfunction of phagocytic function (9). Their cholesterol efflux is positively correlated with tumor burden. Recent studies have shown that mesenchymal like glioblastoma cells heavily rely on local cholesterol metabolism, and TAMs with lipid overload (long-term exposure to myelin debris) provide necessary energy substrates for tumor survival/proliferation (94). Enhanced cholesterol efflux enhances IL-4 signaling and suppresses IFN gamma responsive genes in macrophages, promoting tumor progression (95). Therefore, targeting the mechanism of cholesterol efflux represents a potential anti-cancer strategy (35, 96).

It is worth noting that the metabolic reprogramming of TAMs caused by chemotherapy/radiotherapy may abnormally lead to immune therapy resistance (97). The accumulation of lipid droplets in TAMs has a dual pro-tumor effect-promoting tumor cell proliferation while enhancing immune suppression through M2-like polarization. Targeting pathways such as CD36-mediated fatty acid uptake, FABP4/5 transport, and ABCA1/G1 cholesterol efflux may simultaneously disrupt the metabolic interaction between tumors and TAMs, and alleviate immune suppression, providing a promising strategy for combination immunotherapy.

### 4.4 Amino acid metabolism

In addition to characteristic changes in glucose and lipid metabolism, increasing evidence suggests that the amino acid metabolism pathways of TAMs have also undergone significant remodeling, with glutamine (Gln) metabolism playing a particularly critical role in maintaining their pro-tumor function. In TAMs biology, glutamine is the most widely studied amino acid. It is synthesized by glutamine synthetase (GS) and is an important metabolic substrate for various biosynthesis processes, including nucleotide synthesis, lipid synthesis, and protein production. It can also promote gluconeogenesis, TCA cycle supplementation, and glutathione (GSH) metabolism (98, 99).

Mechanism studies have shown that metabolites derived from glutamine have different effects on the polarization state of macrophages: drug treatment that inhibits GS activity can cause macrophages to transition from M2-like to M1-like with anti-tumor effects, thereby inhibiting the metastasis process (100–102). The glutamine metabolic pathway produces some intermediates, such as lactate and alpha ketoglutarate (α-KG), which can actively promote M2-like cell polarization. Meanwhile, the low oxygen state and elevated GSH levels accompanying TME synergistically inhibit M1-like cell polarization through redox mechanisms (103). These findings have inspired new therapeutic strategies, particularly through nanoparticle-mediated glutamine metabolism targeted therapy, which can effectively prevent M2-like cell polarization and enhance anti-tumor immune response by disrupting TAMs metabolic adaptation, regulating epigenetic regulation dependent on α-KG, and reducing GSH to restore M1-like tumor surveillance (104, 105). The complex interaction between glutamine metabolism and TAMs polarization highlights its potential as a therapeutic target to overcome immune suppression in the microenvironment and enhance the efficacy of immunotherapy.

The metabolic fate of arginine is a factor in regulating the phenotype polarization of TAMs, and different catabolic pathways promote functional differences between M1-like and M2-like subgroups. In M1-like TAMs, inducible nitric oxide synthase (iNOS)-mediated arginine metabolism produces a large amount of nitric oxide (NO), which is a effector molecule that not only has direct anti-tumor effects, but also enhances M1-like polarization through autocrine and paracrine signals (102, 106, 107).By contrast, M2-like TAMs demonstrate pronounced ARG1 upregulation, channeling arginine flux into ornithine and polyamine production. These metabolites establish immunosuppressive conditions and consolidate the M2-like phenotype (108–110). In gliomas, this metabolic interplay is particularly apparent: arginine limitation suppresses TAMs ARG1 expression, thereby restoring pro-inflammatory and phagocytic functions (106). The ARG1-iNOS axis in arginine metabolism constitutes a pivotal switch controlling TAMs polarization. Simultaneous iNOS activation and ARG1 inhibition can repolarize TAMs from M2-like immunosuppressive to M1-like anti-tumor states. This metabolic intervention potentiates NO-based anti-tumor immunity while curtailing polyamine-driven immunosuppression, representing a promising avenue to circumvent immunotherapy resistance mechanisms.

### 4.5 Impact of hypoxia on TAMs

Hypoxia can promote dual interaction between tumors and TAMs in TME, thereby exerting a dual effect of promoting tumor growth and inhibiting tumor growth. Hypoxia can shift the metabolism of tumors from OXPHOS to glycolysis, and also recruit TAMs similar to M2-like type to hypoxic areas, thereby enhancing tumor invasiveness and treatment resistance (111–113). On the contrary, M1-like TAMs tend to cluster in aerobic regions (114, 115), revealing heterogeneity in spatial functionality. This hypoxia TAMs axis forms a self-sustaining pro-tumor ecosystem while providing targeted metabolic immune weaknesses.

Hypoxia shapes TAMs polarization by inhibiting M1-like cell populations and their secreted pro-inflammatory cytokines (IL-1β, TNF-α, CCL17), and promotes M2-like differentiation through microRNA-30c-mediated mTOR/glycolysis inhibition and exosome dependent interactions (116–119). On the contrary, reducing hypoxia can reposition TAMs to the M1-like phenotype, enhancing the efficacy of immunotherapy (117, 120–122), and positioning oxygen regulation as a promising strategy to disrupt the immunosuppressive hypoxic microenvironment.

**Figure 3:**
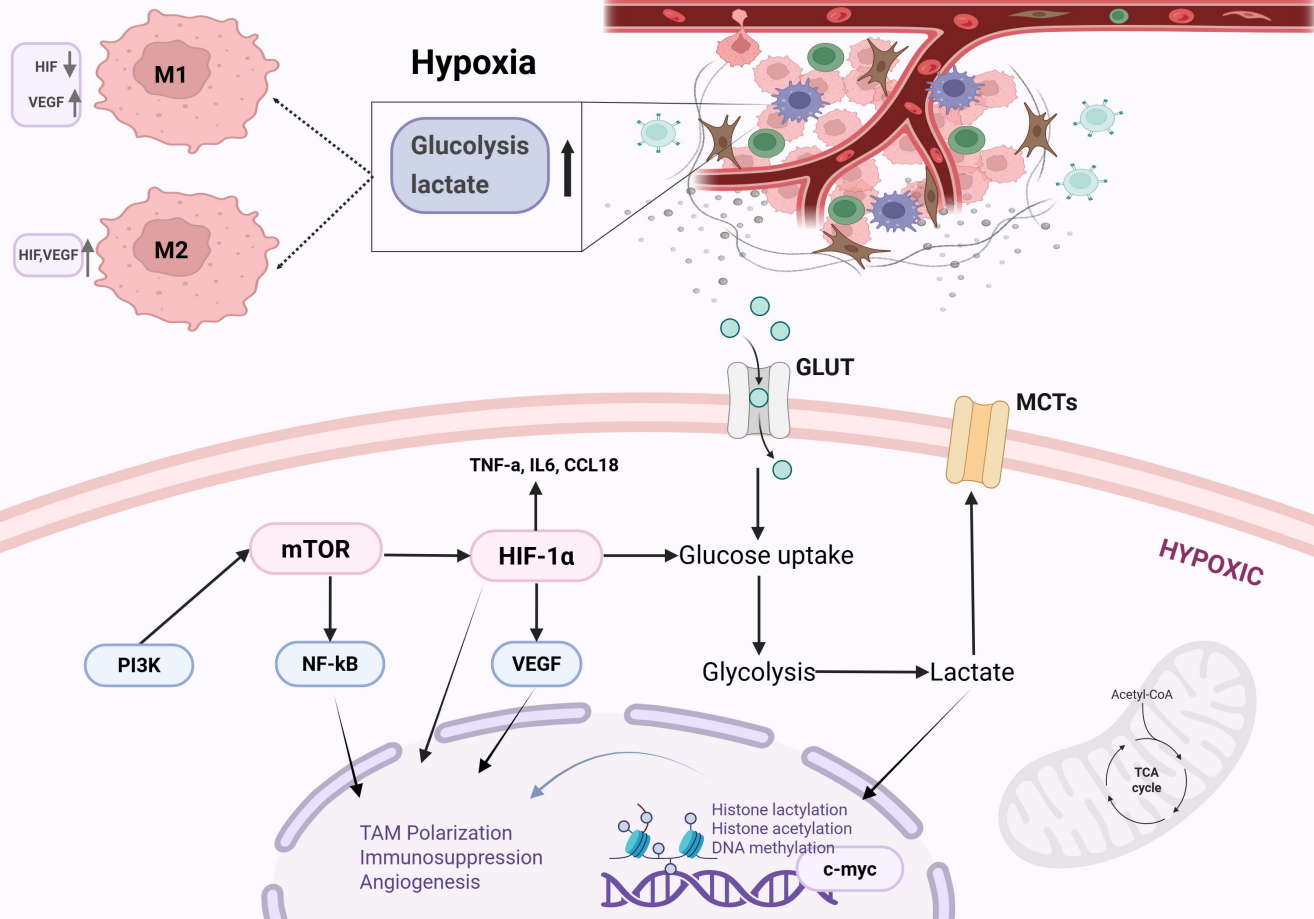
Hypoxia and lactate synergistically promote TAM-mediated immunosuppression via HIF-1α/mTOR signaling. Hypoxia promotes TAM polarization through HIF-1α-mediated metabolic reprogramming. mTOR influences this process by activating the transcription of HIF-related genes, thereby impacting cellular metabolism. Additionally, lactate can stabilize HIF-1α and enhance the expression of VEGF, arginase-1 (Arg1), and other M2-associated genes in TAMs.

The response of cells to hypoxia is mainly mediated by hypoxia inducible factors (HIFs), among which HIF-1α and HIF-2α dominate. HIF-1α, as the main transcription regulator, enhances the glycolytic ability of TAMs by upregulating glucose transporter 1/3 and glycolytic enzymes, while regulating the accumulation of metabolites including lactate, pyruvate, glutathione, and NADPH (118, 123). In addition to glycolysis, activation of HIF-1α also stimulates the serine synthesis pathway and pentose phosphate pathway, promoting the redistribution of ATP within cells, which is crucial for the migration ability of TAMs (66, 124). Hypoxia stabilizes HIF-1α by inhibiting prolyl hydroxylases (PHD), while the PI3K/mTOR pathway enhances HIF-1α expression (125, 126). Together, these events upregulate glucose uptake and glycolysis, leading to the accumulation of lactate—produced by both tumor cells and TAMs—in the tumor microenvironment (**Figure 3**).

Meanwhile, overexpression of HIF-1α in TAMs promotes the production of VEGF-A, thereby promoting tumor angiogenesis (127). In contrast, the expression of HIF-2α preferentially occurs in TAMs induced by hypoxia related cytokines such as colony-stimulating factor-1 (CSF-1) and interleukin-6 (IL-6), where it specifically promotes M2-like polarization (128), It is worth noting that pharmacological inhibition of HIF-2α leads to an increase in microglia in the TAMs population, which is positively correlated with improved patient survival (129). In summary, hypoxia concurrently modulates various metabolic pathways through HIF-1α and HIF-2α, directly or indirectly driving tumor cell migration, angiogenesis, and M2-like TAM polarization.

Hypoxia can drive TAMs-mediated immune suppression through adrenomedullin (ADM) signaling, disrupt endothelial cell connections, lower levels of tight junction proteins (such as Claudin-5, ZO-1), and impair drug delivery (113). Blocking ADM can restore vascular integrity, reverse the immunosuppressive effect of TAMs, and enhance chemotherapy efficacy in preclinical models (130), providing a dual vascular normalization and immune regulation strategy to combat hypoxic tumors.

Hypoxia drives the polarization of M2-like TAMs towards a pro-tumorigenic phenotype through HIF dependent signaling, metabolic reprogramming, and lactic acidosis, thereby achieving tumor promoting functions (PD-L1/IL-10-mediated immunosuppression, VEGF/ADM induced angiogenesis, MMP dependent extracellular matrix remodeling). Therapeutic hypoxia regulation (oxygen supply, HIF inhibition, vascular normalization) can convert TAMs back to the anti-tumor M1-like phenotype, which may work synergistically with immunotherapy to overcome immune suppression and restore T cell response.

### 4.6 TCA cycle intermediates

#### 4.6.1 Succinate

The Warburg effect in tumors can lead to the accumulation of succinic acid, which is caused by impaired TCA cycle (131, 132), which activates succinic acid receptor 1 (SUCNR1) on TAMs. SUCNR1, a G protein-coupled receptor, functions as a key metabolic sensor that translates this accumulation into pro-tumorigenic signals. This triggers the PI3K/AKT/HIF-1α signaling pathway, promoting M2-like polarization, angiogenesis, and metastasis (133), forming a positive feedback loop that promotes immune escape.

Low oxygen environment induces a large accumulation of succinic acid in cells, which is then released from the extracellular environment, making this metabolite a low oxygen biomarker independent of lactate production (134). The accumulation of succinic acid in low oxygen environments activates pro-migration pathways in cancer cells through the stabilization of HIF-1α, thereby enhancing the secretion of IL-1β in TAMs (135). Importantly, this migration phenotype mediated by succinic acid can be effectively eliminated through drug therapy that inhibits HIF-1α (136, 137), indicating that the succinic acid-HIF-1α axis plays a central role in promoting tumor cell proliferation. These findings collectively reveal a low oxygen succinic acid-HIF-1α - IL-1β signaling cascade that links metabolic reprogramming with tumor cell motility and macrophage activation functions in the TME.

As a transcription target of HIF-1α (138, 139), ARG1 is regulated by succinic acid and upregulated in a dose-dependent manner (130), thereby enhancing M2-like polarization. By targeting delivery to regulate the level of succinic acid, TAMs can transition to the M1-like state (136, 140), indicating that succinic acid metabolism is a therapeutic approach that can disrupt tumor promoting TAMs function and enhance immune response.

#### 4.6.2 α-Ketoglutarate

α-Ketoglutarate (α-KG) is produced during D-3-phosphoglycerate dehydrogenase (PHGDH)-mediated serine biosynthesis, serving to maintain the activity of M2-like TAMs via the mTORC1 mechanism (141, 142). PHGDH is the rate-limiting enzyme in the serine biosynthesis pathway. It functions by diverting the glycolytic intermediate 3-phosphoglycerate (3-PG) away from energy production and into the synthesis of serine. Furthermore, the downstream metabolism of serine generates NADPH, a vital cofactor that neutralizes reactive oxygen species (ROS). This antioxidant capacity is required to maintain the survival and immunosuppressive function of M2-like TAMs (141). Exogenous α-KG restores polarization of M2-like macrophages through the action of Jumonji domain protein D3 (JMJD3) (143), while the α-KG/succinic acid ratio determines the stability of HIF-1α (144), thereby positioning α - KG as a metabolic epigenetic regulatory factor that regulates TAMs function.

#### 4.6.3 Adenosine

Adenosine is an immunosuppressive metabolite produced by the breakdown of adenosine triphosphate, which has a dual function: maintaining tissue homeostasis under physiological conditions, and promoting immune escape when accumulated in tumors (145, 146). In the TME, adenosine activates A2A/A2B receptors on TAMs, inducing secretion of IL-10/TGF-β, impaired antigen presentation ability, changes in OXPHOS metabolism, and production of pro angiogenic signals.

Adenosine exerts a multifunctional immunomodulatory effect through differential activation of four G protein coupled receptors (A1, A2A, A2B, and A3), with A2A and A2B subtypes playing a particularly critical role in regulating the polarization and function of TAMs. The adenosine A2A receptor axis not only inhibits anti-tumor immunity by suppressing effector T cell responses (147), but also promotes the pro-tumorigenic activity of TAMs in lung adenocarcinoma by secreting chemotactic and polarizing factors mediated by PI3K/AKT/NF-κB, thereby enhancing the migration and invasion ability of TAMs (148). At the same time, the increase in adenosine deaminase 2 activity in TAMs enhances M2-like polarization, further enhancing its ability to promote tumors (149). The hypoxic TME exacerbates adenosine accumulation by increasing the breakdown of ATP, forming a positive feedback loop that maintains immunosuppressive status through polarization of M2-like TAMs (145). In addition, adenosine signaling upregulates the expression of thromboreactive protein-1 (TSP-1) in macrophages to stimulate angiogenesis, while activation of A2A receptors simultaneously inhibits TLR-mediated production of tumor necrosis factor - α (TNF-α) and enhances the expression of VEGF (150). Collectively, these mechanisms establish adenosine as the predominant modulator of TAMs function, synchronously regulating immune evasion, angiogenic processes, and metabolic adaptation in the TME.

The tissue-specific effects of adenosine signaling—cytoprotective in normal contexts versus oncogenic in tumors—highlight the rationale for selectively targeting its immunosuppressive components. Adenosine promotes M2-like TAMs polarization, contributing to tumor growth and immune escape. Antagonizing adenosine-receptor binding can reprogram TAMs toward an M1-like anti-tumor phenotype, strengthening anti-tumor immunity via improved antigen presentation and T-cell priming (151). The combination of adenosine pathway inhibitors with conventional immunotherapy constitutes a promising therapeutic strategy.

### 4.7 Metabolic crosstalk and integrated regulatory networks in TAMs

TAMs are governed by the synergistic regulation of multiple metabolic pathways, encompassing glucose and lipid metabolism, amino acid catabolism, mitochondrial bioenergetics and purine metabolism (152–154). However, these pathways do not operate in isolation, rather, they function within a complex, interwoven regulatory network characterized by extensive crosstalk.

First, a critical convergence exists between glucose and lipid metabolism. Glycolytic intermediates, notably acetyl-CoA, serve as essential precursors for *de novo* lipogenesis. Conversely, lipid-derived signaling molecules—such as oxidized lipids and lipid second messengers—exert retrograde regulation on the activity and expression of rate-limiting glycolytic enzymes, establishing a bidirectional control circuit (9). Furthermore, the glycolysis-cholesterol axis has emerged as a pivotal node for immune modulation within the tumor microenvironment. This axis functions as a metabolic signaling network, where glycolytic intermediates directly fuel cholesterol biosynthesis to dictate macrophage phenotype. Consequently, it exerts a dualistic influence, simultaneously supporting tumor progression while potentially constraining antitumor immune responses (155).

Second, the HIF-1α signaling pathway plays a central role in orchestrating these integrated metabolic programs. Evidence indicates that HIF-1α not only drives M2-like polarization but also engages in functional crosstalk with lipid regulatory factors, such as PPARγ, to co-drive triglyceride synthesis and inflammatory responses (156, 157). Beyond canonical transcriptional regulation, HIF-1α is subject to non-transcriptional modulation; for instance, activation via the IL-15 receptor complex or the IL-18/IL-18R1 axis can stabilize HIF-1α, thereby modulating the expression of the chemokine CX3CL1 and mediating critical communication between macrophages and tumor cells (154, 158, 159).

In summary, metabolic regulation in TAMs constitutes a multi-layered, dynamic, and highly interconnected process. Future research must prioritize elucidating the molecular mechanisms governing these nodal points and evaluating their potential as therapeutic targets for immunometabolic intervention.

## 5 Targeting TAMs metabolism for cancer therapy: from mechanism to clinical application

In recent decades, four main treatment strategies have emerged for cancer treatment targeting TAMs: (1) phenotype reprogramming from a pro-tumor state to an anti-tumor state, (2) selective clearance of TAMs, (3) blockade of monocyte recruitment, and (4) development of chimeric antigen receptor (CAR) - expressing macrophages (CAR Macs) (160, 161). This article focuses on introducing cutting-edge interventions targeting the biological metabolic pathways of TAMs, emphasizing strategies to enhance anti-tumor immunity by utilizing the unique metabolic weaknesses of these cells.

### 5.1 Targeting glycolysis in TAMs

The glycolysis inhibitor 2-deoxy-D-glucose (2-DG) can block the regulatory enzyme hexokinase activity of glycolysis and has been shown to effectively inhibit M1-like polarization of TAMs (65). It is worth noting that preclinical studies have shown that the combination of low-dose 2-DG and MEK inhibitors can synergistically promote apoptosis of pancreatic ductal adenocarcinoma cells. This combination therapy inhibited tumor growth in xenograft models and prolonged the survival time of experimental animals (51, 127, 162). Targeting glycolysis has also been shown to potentiate the efficacy of existing immunotherapies. The innovative method of using injectable hot gel containing both GLUT1 inhibitor and PD-1/PD-L1 blocker also showed good effect in the glioblastoma (GBM) model. In this case, dual metabolism/immune regulation can delay tumor progression and prolong survival time (163).

targeting glycolysis in TAMs not only directly attenuates their tumor-promoting functions but also synergistically enhances the efficacy of radiotherapy, chemotherapy, and immune checkpoint inhibitors (164). This is achieved through mechanisms such as modulating lactate levels, alleviating microenvironmental acidosis, and potentiating T cell activity.

### 5.2 Lactate targeting in TAMs

Recent preclinical studies have also focused on lactate metabolism, viewing them as targets for regulating immunosuppressive TME. Inhibition of lactate production has been proven to simultaneously reduce lactate levels and inhibit malignant tumor development. Currently, multiple methods are being studied to reverse the polarization of TAMs from immunosuppressive M2-like type to tumor killing M1-like type (68, 165, 166).In a breast cancer model, liposomes co-loaded with lonidamine and sirocinib suppress glycolysis and MCT-4 expression, thereby reducing lactate efflux and promoting the polarization of TAMs toward the M1-like phenotype (167).

Lactate-mediated immunosuppression severely limits the efficacy of immune checkpoint inhibitors, whereas targeting lactate metabolism enhances their therapeutic potential. Inhibition of MCT1 or LDHA boosts anti-PD-1 efficacy by 1622-fold (168); in glioblastoma, combined targeting of lactate transport and lactylation of the DNA repair protein KU70 synergistically suppresses tumor growth (169). Furthermore, 1C8, an inhibitor targeting serine/arginine-rich splicing factor 10 (SRSF10), overcomes resistance to PD-1 blockade in hepatocellular carcinoma (73).

The translational progress of this strategy is summarized in **Table 1**, which details small-molecule inhibitors and modulators currently under clinical investigation. As shown, therapeutic efforts have diversified across multiple nodes of lactate metabolism: from LDHA inhibitors (PX-478, GSK2837808A) that block lactate generation, to MCT1/4 blockers (AZD3965, syrosingopine) that prevent lactate efflux, and novel agents targeting the lactate receptor GPR81. Notably, several of these agents have advanced to Phase I/II clinical trials (identified by NCT numbers in **Table 1**) across various malignancies, demonstrating preliminary evidence of metabolic modulation and immune activation. However, despite this promising clinical pipeline, challenges remain regarding the systemic toxicity of blocking ubiquitous metabolic pathways and the potential for compensatory metabolic plasticity (**Table 1**).

**Table 1:** Clinical development of lactate metabolism modulators targeting TAMs: Targets, mechanisms, and trial status.

Molecular target	MoA	Targeted medicine	Effect on tams	NCT no.	Clinical phase
HIF-1α	Promotes angiogenesis, glycolysis, and invasion	PX-478	Reduced PD-L1 expression and reversed TAMs M2 polarization	I	NCT00522652
LDHA	Simultaneously inhibits Bcl-2 (pro-apoptotic) and LDHA/LDHB (inhibits metabolism	AT-101	By inhibiting lactate production, it indirectly improves the immunosuppressive environment.	II	NCT00286780
PKM2	The key enzyme in the last step of glycolysis, its isoform PKM2, promotes lactate production	Shikonin	Promote tumor cell proliferation and metastasis, and increase the number of tumor-specific T cells	I	NCT01968928
MCT1	Lactic acid in the TME is taken as fuel for oxidative metabolism	AZD3965	Blocks lactic acid intake, leads to lactic acid accumulation, and inhibits glycolysis	I	NCT01791595
NF-κB	Glycolytic metabolism is promoted by activating the NF-kB signaling pathway, leading to increased lactate production	Curcumin	The expression of FoxP3 in Tregs was down-regulated, and the transformation of TAMs into M1 type was promoted	II	NCT00094445
TGF-β	Lactylation modification of Snail1 activates the TGF-β/Smad2 signaling pathway by promoting its nuclear translocation	Galunisertib	Tams secrete TGF-β→ promote Treg recruitment and form immunosuppressive TME	II	NCT02452008
PDK1	Relieves inhibition of PDCs, restores pyruvate into mitochondria, and reverses the Warburg effect	TQB3525	It relieves the inhibition of cytotoxic T cells and NK cells, inhibits the polarization of M2 macrophages and the activity of MDSCs	I/II	NCT04690725
MCT4	It is responsible for rapidly excreting lactic acid produced in highly glycolytic tumor cells and CAFs into the TME to avoid intracellular acidosis	Metformin	Remove lactic acid, significantly improve TME, and disrupt metabolic symbiosis	I	NCT02083692

Lactic acid has a conditionally dependent effect on macrophage function: in hepatocellular carcinoma, D-lactate enhances the phagocytic ability of Kupffer cells by inhibiting the PI3K/Akt pathway and activating NF-κB, thereby promoting M1-like polarization phenotype (170). In contrast, in PTEN/p53 deficient prostate cancer, tumor-derived L-lactic acid induces histone lactylation in TAMs, enhancing immunosuppressive M2-like polarization (171).

### 5.3 Inhibiting fatty acid oxidation

Inhibition of FAO has been shown to effectively reverse the immunosuppressive phenotype of TAMs and impede tumor progression. For instance, in colorectal cancer, lactate secreted by tumor cells upregulates VSIG4 expression in macrophages via the H3K18la-METTL14-m6A axis. This subsequently activates the JAK2/STAT3 signaling pathway, promoting FAO and PPAR-γ expression, thereby driving M2-like polarization. Targeting VSIG4 or inhibiting FAO can reverse this process, suppressing tumor growth and enhancing the therapeutic efficacy of PD-L1 blockade (172).In non-small cell lung cancer (NSCLC), Semaphorin 7A promotes the polarization of TAMs toward the M2-like phenotype via an ITGB1-dependent pathway, a process in which FAO plays a pivotal role (173).

In addition to inhibiting mTOR, strategies targeting lipid metabolism have also shown significant potential, including CSF1R inhibitors that can reduce TAMs cell populations and CCR2/CCR5 antagonists that can block monocyte recruitment, many of which have entered clinical trial (174, 175). Cholesterol regulators such as lipostatin can not only inhibit TAMs-mediated epithelial mesenchymal transition, but also synergize with anti-PD-1 therapy to enhance the effect of immune checkpoint blockade (176, 177).

### 5.4 Modulating amino acid metabolism

Targeting amino acid metabolism in TAMs has emerged as a pivotal strategy for cancer immunotherapy. For instance, in colorectal cancer, the deficiency of amino acid metabolic enzymes, such as acyl-CoA dehydrogenase short chain (ACADS), within TAMs facilitates their polarization toward the M2-like phenotype (178). In prostate and bladder tumors, the glutamine antagonist JHU083 reprograms TAMs, inducing a pro-inflammatory phenotype, enhancing phagocytic capacity, and inhibiting angiogenesis, ultimately leading to tumor cell apoptosis (179).

Targeting amino acid metabolic pathways, specifically arginine and tryptophan, has emerged as an effective strategy for modulating TAMs polarization. Decitabine (a DNA methyltransferase inhibitor) coordinately downregulates ARG1 and upregulates iNOS through RIPK3-dependent signaling, driving TAMs conversion to the M1-like phenotype. Etomoxir, a FAO inhibitor, similarly augments anti-tumor immunity via iNOS upregulation (106, 110, 180, 181). These comprehensive metabolic reprogramming approaches collectively herald a new era in cancer therapy, presenting viable strategies to circumvent microenvironment-induced immunosuppression and optimize treatment responses in diverse malignancies.

### 5.5 Targeting tumor hypoxia

The latest evidence from preclinical and clinical studies suggests that reversing hypoxia through oxygen supply strategies can convert TAMs back into anti-tumor phenotypes, thereby restoring immune surveillance and enhancing immunotherapy efficacy (182–184). Multiple HIF inhibitors targeting tumor hypoxia have been developed, which exert their effects through different mechanisms. The combination of HIF-1α inhibitor PX-478 and immune checkpoint blockade has shown good anti-tumor effects, reducing EMT and enhancing treatment response (185, 186). The nano delivery mode of HIF-1α inhibitor FB15 can specifically inhibit the growth of breast cancer in the hypoxic area (187). In clinical practice, the HIF-2α inhibitor belzutifan approved by the US Food and Drug Administration (FDA) has become an important means of treating VHL related tumors, including renal cell carcinoma and pancreatic neuroendocrine tumors (115). These advances demonstrate the therapeutic potential of HIF inhibition in cancer treatment.

During tumorigenesis and cancer progression, the mTOR signaling pathway directly regulates the metabolic reprogramming of TAMs at multiple levels. MTOR activation promotes M2-like polarization by upregulating p-STAT3 and IL-10 (188). Rapamycin is a specific mTORC1 inhibitor with dual anti-tumor effects. On the one hand, it can directly inhibit the proliferation of cancer cells, and on the other hand, it can promote the polarization transformation of TAMs towards M1-like phenotype with anti-tumor effects (189, 190).

### 5.6 Challenges and future perspectives

Although targeting TAMs metabolism holds immense therapeutic potential, its clinical translation is impeded by multifaceted systemic challenges. Biologically, the heterogeneity of TAMs origins, dynamic phenotypic switching, and distinct spatial distributions contribute to unstable therapeutic efficacy (155, 191). Moreover, TAMs metabolic states are subject to real-time modulation by microenvironmental cues, fostering adaptive reprogramming that enables evasion of intervention (192).

Beyond these biological barriers, a critical pharmacological hurdle lies in the lack of tumor-macrophage selectivity. As summarized in **Table 2**, while a broad spectrum of metabolic inhibitors (e.g., glycolysis inhibitors like 2-DG and BAY-876; glutaminolysis inhibitors like CB-839) can effectively reprogram TAMs, they target pathways fundamental to all rapidly dividing cells, including activated effector T cells and normal tissue progenitors (193). Consequently, systemic administration risks inducing severe immunosuppression by impairing anti-tumor cytotoxic T lymphocyte (CTL) function or causing dose-limiting toxicities. A striking example is HK2 inhibition: while it successfully reverses the pro-metastatic properties of TAMs in pancreatic cancer models, it simultaneously starves CTLs—which also rely heavily on glycolysis—creating a significant therapeutic paradox (194–196) (**Table 2**).

**Table 2:** Summary of metabolic inhibitors targeting TAMs: Mechanisms, cancer types, and reprogramming effects.

Metabolic targeting		Targeted medicne	Cancer type	Effect on TAMs	PMID
Glucose metabolism		HK2 (2DG)	Pancreatic cancer	Inhibit glycolysis in TAMs to reverse their pro-metastatic properties.	34198548
		HK(Quercetin)	Breast cancer	Suppress the infiltration of M2-like TAMs.	37587146
		HK(3-Bromopyruvate)	Colorectal cancer	Promotes the polarization of tumor-associated macrophages (TAMs) towards an anti-tumor M1 phenotype.	38862461
		GLUT1(BAY-876)	Glioblastoma	Decrease the population of immunosuppressive TAMs within the tumor microenvironment.	38460167
		LDHA(FX11)	Prostate Cancer	Suppress the infiltration of M2-like TAMs.	38831751
		PARP enzyme(Olaparib)	Breast cancer	Inhibit the polarization of macrophages into TAMs.	38802355
		PARP enzyme(Talazoparib)	Colorectal cancer	Reprogram tumor-associated macrophages (TAMs) into a more antitumor phenotype.	37702298
		PARP enzyme(Lonidamine )	Cancer	Alleviate tumor microenvironment (TME) acidity to prevent the immunosuppressive polarization of M2 macrophages.	37272777
		HIF-1α(PX-478)	Lung cancer	Inhibit the polarization of macrophages into TAMs.	37478668
		HIF-1α(Acriflavine)	Glioma	Prevent the immunosuppressive polarization of M2 macrophages.	27602954
		HIF-1α(YC-1)	Triple-negative breast cancer	Shift TAM polarization from the M2 to M1 phenotype to inhibit angiogenesis.	36822023
		mTORC(Rapamycin)	Glioblastoma	Induce selective apoptosis of M2-polarized tumor-associated macrophages.	32020472
Amino acid metabolism		IDO1(Epacadostat)	NSCLC	Inhibit the M2 polarization of TAMs.	37187591
		Arginine(Sepiapterin)	Breast tumor	Enhance the expression of markers associated with intratumoral M1-polarized tumor-associated macrophages (M1-TAMs).	39191486
		Arginine(Piceatannol 3′-O-glucoside)	Colorectal cancer	Suppress M2-like macrophage populations and concurrently induce polarization toward the M1-like phenotype.	39375706
		GLS(CB-839)		Block the differentiation of M0 macrophages into immunosuppressive M2-like macrophages.	33796407
		GLS(BPTES)	Cholangiocarcinoma	Promote M1 macrophage polarization and reduce M2 macrophage levels.	38837691
		SLC7A11(Erastin)	Nasopharyngeal carcinoma	Facilitate the polarization of macrophages from the M2 to M1 phenotype.	37451020
		SLC7A11(Sulfasalazine)	Hepatocellular carcinoma	Induce repolarization of macrophages into an M1-like phenotype.	37132587
Lipid metabolism		LXR(TO901317)	Lung cancer	TAMs exert broad detrimental effects through various mechanisms that sustain an immunosuppressive microenvironment.	33361391
		HMG-CoA( Simvastatin)	Glioma	Affects the lipid metabolism of malignantly transformed macrophages, and subsequently inhibits its proliferation, migration, and invasion ability.	36274598
		HMG-CoA ( Lovastatin)	Head and neck squamous cell carcinoma	Shifts from M2 to M1 macrophage predominance	36725087
		HMG-CoA(Betulinic Acid)	Gastric cancer	Inhibit the polarization of macrophages into TAMs and suppress TAM-mediated tumorigenicity.	36838713

HK, Hexokinase; GLUT1, Glucose Transporter 1; LDHA, Lactate Dehydrogenase A; AMPK, AMP-Activated Protein Kinase; PARP, Poly (ADP-Ribose) Polymerase; HIF-1α, Hypoxia-Inducible Factor 1-alpha; mTORC, Mechanistic Target of Rapamycin Complex; IDO1, Indoleamine 2;3-Dioxygenase 1; GLS, Glutaminase; SLC7A11, Solute Carrier Family 7 Member 11; LXR, Liver X Receptor; HMG-CoA, 3-Hydroxy-3-Methylglutaryl-Coenzyme A; NSCLC, Non-Small Cell LungCancer; 2DG, 2-Deoxy-D-glucose; YC-1, 3-(5'-Hydroxymethyl-2'-furyl)-1-benzylindazole; BPTES, Bis-2-(5-phenylacetamido-1;3; 4-thiadiazol-2-yl) ethyl sulfide.

Insufficient targeted delivery efficiency remains a critical bottleneck for clinical translation. Free drugs suffer from inherent limitations such as poor solubility, suboptimal pharmacokinetics, and a lack of cell specificity (197). Although nanocarriers offer improved delivery capabilities, they still face significant challenges, including the scarcity of specific targets and the difficulty in penetrating physiological barriers like the blood-brain barrier (198).

Although targeting the metabolic reprogramming of TAMs holds immense therapeutic potential, its clinical translation remains constrained by significant bottlenecks. Future research must establish a paradigm of precise spatiotemporal dynamic intervention: on one hand, focusing on disrupting metabolic symbiotic networks by developing multifunctional nanosystems capable of co-delivering photosensitizers, nanozymes, and siMCT4; on the other hand, promoting combined interventions across multiple metabolic pathways, or synergizing with immune checkpoint inhibitors to enhance therapeutic efficacy (199–201).

## 6 Conclusion

Within the TME, TAMs undergo extensive metabolic rewiring, establishing a pro-tumor phenotype that facilitates tumor progression, immune evasion, and therapeutic resistance. This metabolic shift encompasses coordinated alterations in glucose, lipid, and amino acid metabolism, collectively reinforcing M2-like polarization and immunosuppressive activities. The metabolic crosstalk between TAMs and malignant cells creates a positive feedback circuit that sustains tumor growth while dampening anti-tumor immunity. Recent investigations into these metabolic pathways have uncovered promising therapeutic avenues to modulate TAMs function and counteract microenvironment-driven immune suppression. Emerging approaches prioritize disrupting critical metabolic checkpoints to reverse TAMs polarization, potentiate immune checkpoint blockade efficacy, and restore anti-tumor responses, offering novel combination therapy strategies for oncology treatment. Metabolic reprogramming regulates TAMs polarization. Strategies include inhibiting glycolysis, lactate transport, lipid uptake, and amino acid metabolism. Complementary methods alleviate hypoxia to repolarize TAMs to M1-like type. Combining these with immunotherapy synergistically overcomes suppression, linking metabolic regulation with immune enhancement.

However, the translational journey from preclinical discovery to clinical application remains fraught with challenges. The remarkable efficacy of metabolic reprogramming strategies in murine models often fails to translate into consistent benefits in human patients, a discrepancy largely attributable to the greater complexity and heterogeneity of the human tumor microenvironment. Furthermore, the therapeutic outcomes are likely to be highly context-dependent, varying not only across distinct tumor types but also spatially within individual tumors—where metabolic phenotypes can differ markedly between the hypoxic core and the invasive front. This inherent variability underscores a critical limitation: a one-size-fits-all approach to TAMs metabolism targeting is unlikely to succeed. Therefore, while the metabolic reprogramming of TAMs holds immense therapeutic promise, its successful clinical implementation demands a paradigm shift toward more nuanced, context-aware, and personalized strategies. Achieving this will require not only continued fundamental research into the dynamic interplay between metabolism and immunity, but also the development of sophisticated diagnostic tools—such as non-invasive imaging probes capable of mapping real-time metabolic activity—and the execution of well-designed clinical trials that incorporate longitudinal, multi-omic profiling of the TME.

In conclusion, metabolically targeted modulation of TAMs offers a paradigm-shifting therapeutic strategy for oncology, capable of reversing immunosuppressive TMEs and addressing current immunotherapy limitations. This approach can reprogram tumor-promoting M2-like TAMs into anti-tumor M1-like phenotypes, generating renewed optimism for treating advanced malignancies and potentially transforming the management of therapy-resistant cancers through combinatorial regimens.
